# Notes and News

**Published:** 1902-04-05

**Authors:** 


### Notes and News.

Three cases of small-pox have occurred at Godalming and one at Aylsham.

According to Dr. Wutzdorff of the German Imperial Board of Health, cancer has increased in Germany 18·5 per cent. during the last six years.

Medical tours to health resorts are growing in favour. There is a society in Germany and Austro-Hungary for the promotion of these tours, and in September next its members will visit several health resorts, concluding with Carlsbad, where the Congress of German Scientists and Medical Practitioners will hold a congress this year.

Many persons are under the impression that vaccination is of no use after contagion. The report of the Liverpool Medical Officer of Health contains evidence against this theory. He gives a case in which a number of persons in a lodging house where small-pox occurred were all vaccinated save thirteen. All the vaccinated escaped the disease, whilst of the thirteen unvaccinated persons nine contracted it.

Liverpool charities have largely benefited through the will of the late Miss Olivia Atherton. She has left £1,000 each to the Royal Infirmary, the David Lewis Northern Hospital, the Southern Hospital, and the Liverpool Country Hospital for Chronic Diseases. To the Liverpool Hospital for Women, the Eye and Ear Infirmary, the Liverpool dispensaries, the Children's Infirmary, the Bootle Hospital, and the Stanley Hospital, she has left £500 each, and to other medical charities £250 each.

It will be interesting to see whether the testimony of M. Leopold Perrin, of the *Suisse Liberale*, who has been visiting the concentration camps in South Africa, will cause any change of public opinion in Switzerland. We fear not. That country accepted the falsehoods circulated against us with joyful avidity, and it is not likely they will abandon cherished beliefs. M. Perrin found nothing to complain of, and in the hospitals connected with the camps he notes that every luxury necessary for the patients was given to them. In one case he reports that two bottles of champagne were provided in one day.

The Poplar Board of Guardians recently appointed Miss Lamport, a lady doctor, as public vaccinator. This is the first appointment in England of a woman to this office. It is hoped that the arrangement will encourage a larger number of females to submit themselves for vaccination.

The sect known as the Peculiar People continue to disregard the warnings they receive, and run the risk of conviction for manslaughter, by neglecting to secure medical aid for their sick. At Ilverton, recently, a child belonging to these people died of pneumonia, no doctor was called in, and the medical certificate stated that with treatment the malady could have been alleviated if not cured.

The Local Government Board is taking strong measures to secure, as far as they are concerned, the proper administration of the laws relating to vaccination. Realising that the real responsibility of prosecution rests with the vaccination officers, they are dismissing these officers when they find them neglectful and injudicious in the conduct of their duties. This action on their part is likely to stimulate activity.

A meeting of the visiting committee of King Edward's Hospital Fund was held last week at St. Thomas's Hospital in the committee-room, at which a large number of medical and lay members of the committee were present. A letter was read from Sir Arthur Bigge to the honorary secretaries as follows:—"The Prince of Wales directs me to ask you to be good enough to convey to the visiting committee of King Edward's Hospital Fund the expression of his gratification at the increase in their numbers. His Royal Highness feels that this arrangement will give still more strength and importance to the committee. His Royal Highness appreciates to the fullest extent the valuable work which has been done by the committee, and looks forward with confidence to ever increasing good results from their labours." Eighty-eight hospitals which had applied were divided into 22 groups among the visitors, each hospital to be visited by a medical man and a layman.

The fourteenth International Medical Congress will take place next year in Madrid during the month of April, and arrangements are already in progress for making the meeting a success, and there is no doubt that Madrid will be found a very pleasant place to which to make an outing. The honorary secretaries of the National Committee for Great Britain and Ireland inform us that the present rate of exchange makes it worth while for those who propose to attend the Madrid meeting to pay their subscriptions at once, as the value of the peseta varies from time to time. The necessary forms of application for membership can be obtained from the honorary secretaries, Mr. D'Arcy Power or Dr. Horton-Smith, who are prepared to supply receipts, and cards of membership as soon as they are issued, for postal orders of £1. Ladies, if they accompany members, pay 6s. 8d. for their tickets, which, besides other advantages, give admission to the various fêtes held during the Congress.

